# Vogt-Koyanagi-Harada disease: the step-by-step approach to a better understanding of clinicopathology, immunopathology, diagnosis, and management: a brief review

**DOI:** 10.1186/s12348-022-00293-3

**Published:** 2022-05-12

**Authors:** Cristhian A. Urzua, Carl P. Herbort, Masaru Takeuchi, Ariel Schlaen, Luz E. Concha-del-Rio, Yoshihiko Usui, Loreto Cuitino, Ioannis Papasavvas

**Affiliations:** 1 Laboratory of Ocular and Systemic Autoimmune Diseases, Faculty of Medicine, University of Chile, Santiago, Chile; 2 Department of Ophthalmology, University of Chile, Santiago, Chile; 3 Facultad de Medicina, Clinica Alemana-Universidad del Desarrollo, Santiago, Chile; 4 Retinal and Inflammatory Eye Diseases, Centre for Ophthalmic Specialised Care (COS), Lausanne, Switzerland; 5 Department of Ophthalmology, National Defense Medical College, Tokorozawa, Saitama Japan; 6 Hospital Universitario Austral, Hospital de Clinicas de Buenos Aires, Buenos Aires, Argentina; 7 Inflammatory Eye Disease Clinic, Dr. Luis Sanchez Bulnes Hospital, Asociación para Evitar la Ceguera en México (APEC), Mexico City, CDMX, Mexico; 8 Department of Ophthalmology, Tokyo Medical University, Tokyo, Japan; 9 Servicio de Oftalmología, Hospital Clínico Universidad de Chile, Santiago, Chile

**Keywords:** Chronic VKH, Initial-onset acute VKH, Vogt-Koyanagi-Harada disease, Uveomeningoencephalitic syndrome

## Abstract

**Background:**

Appraisals of Vogt-Koyanagi-Harada disease (VKH) have become progressively more complete, since its first description in 1906. The availability of new investigational methods has improved our knowledge of the immunopathology, clinicopathology, diagnosis, and management of VKH disease. This review aimed to describe some of the steps that led to better characterization of VKH as a clinical entity.

**Methods:**

We searched on PubMed for articles that described the history of VKH disease and analyzed the progress in disease appraisal with new investigational and imaging methods. In particular, we searched for articles that investigated the clinicopathology, diagnosis, and management of VKH.

**Findings:**

The following developments were considered essential for improving the appraisal and understanding of VKH: (1) the history of the disease, (2) immunopathological mechanisms, (3) clinicopathology, (4) the importance of distinguishing initial-onset from chronic disease, (5) relevant imaging modalities, among which indocyanine green angiography is crucial, (6) diagnostic criteria that facilitate early diagnosis, and (7) the need for early, prolonged, aggressive treatment that combines steroidal and non-steroidal immunosuppression.

**Conclusion:**

Based on these findings, the definition of VKH has improved. VKH disease starts in the choroidal stroma and later involves other structures when it is not diagnosed and treated early. Indocyanine green angiography and enhanced depth imaging optical coherence tomography facilitate early diagnosis and precise monitoring of choroidal inflammation. ICGA is clearly the gold standard for appraisals and follow-ups in VKH disease, however EDI-OCT should be especially considered in those areas where ICGA is not fully available. These modalities have contributed substantially to a “cure” for VKH, when treatment is introduced within the therapeutic window of opportunity.

## Introduction

Vogt-Koyanagi-Harada disease (VKH) was first described by Alfred Vogt in 1906 [1, 2]. Vogt’s case description focused principally on eyelash whitening (poliosis) [1]. The article was 14 pages long, and only five lines were devoted to intraocular inflammation [2]. Since that first publication, the appraisal of VKH disease has progressively improved, step by step, up to very recent years. The purpose of this comprehensive global review was to describe the major advancements in the knowledge of VKH disease. VKH disease was defined as “*… a rare, granulomatous inflammatory disease that affects pigmented structures, such as the eye, inner ear, meninges, skin, and hair*” [3]. VKH starts in the eye; specifically, in the melanocyte islets of the choroidal stroma, which leads to the development of primary stromal choroiditis [4]. Failure to treat VKH in the early stage allows the inflammatory process to extend to other ocular structures, including the optic disc and retina; as VKH progresses, granulomatous panuveitis and chronic disease develop [4]. Previously, VKH disease progression was classified as probable (ocular involvement), incomplete (ocular plus integumentary involvement), and complete (ocular plus integumentary plus neurological involvement). Due to the moderate agreement on the diagnostic criteria for VKH and the implications of an early diagnosis on prognosis and disease resolution, our group recently proposed that two distinctive courses can be distinguished in a clinical evaluation of patients with VKH. This proposal was part of a pioneering, pragmatic advancement. These two clinical phenotypes should be identified early; they are known as initial-onset (acute) VKH and chronic (recurrent) VKH [5–7].

This comprehensive review starts from the perspective of a brief historical account; then, we address the more recent advancements in the appraisal of VKH disease. We cover the topics of immunopathology, clinicopathology, the crucial distinction between initial-onset and chronic disease, new imaging modalities, strong and improved diagnostic criteria, and VKH management.

## Brief history of VKH disease

**Fig. 1:**
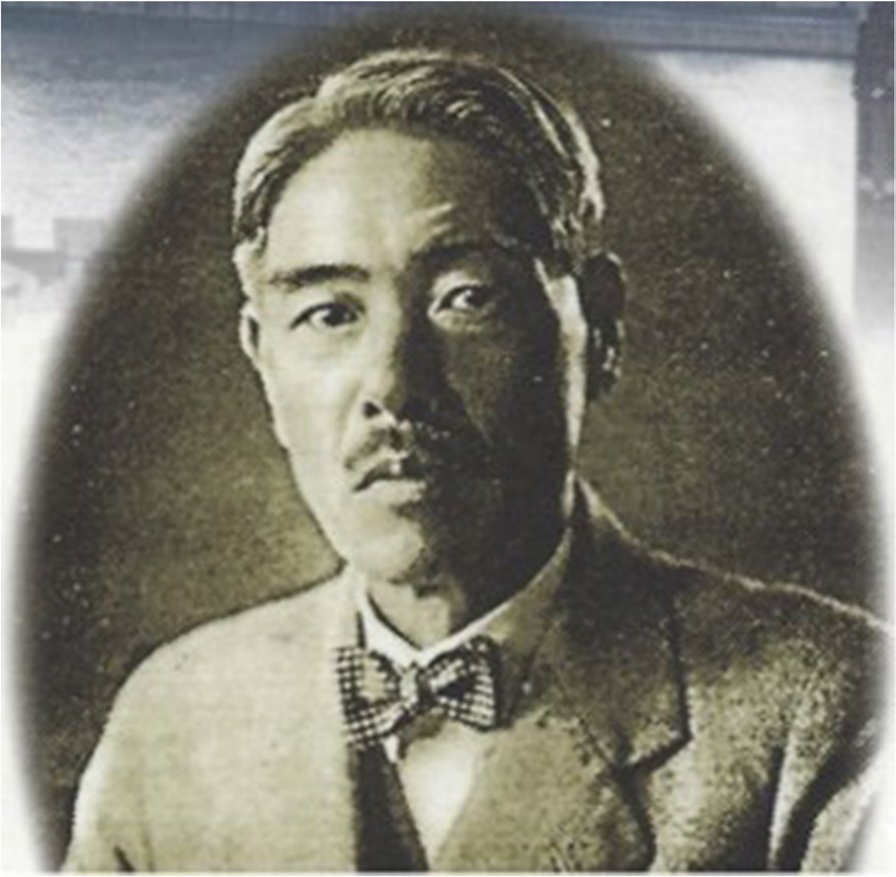
Yoshizo Koyanagi (1880–1954). Yoshizo Koyanagi was born in the Tokyo area in 1880. He graduated from Kyoto Imperial University, one of the seven imperial universities founded after the Meiji restoration and modernization of Japan, at the end of the 19th and beginning of the twentieth century. These universities were established in Tokyo (1886), Kyoto (1897), Tohoku University in Sendai (1907), Kyûshû University in Fukuoka (1911), Sapporo (1918), Osaka (1931) and Nagoya (1939). In 1914, Koyanagi published the first article in Japanese on the disease that would eventually bear his name. He described two patients with all the characteristics of VKH disease. In 1915, he became the first Professor of Ophthalmology of Tohoku Imperial University in Sendai, 300 km north of Tokyo. The lower part of the figure shows the title page of his ground-breaking article, which was published in the Klinische Monatsblätter für Augenheikunde, in the German language. That article described 16 cases of VKH disease, where he revealed the natural course of the disease. At the time, to gain international exposure, Japanese authors mostly published in German journals, because the medical doctrine and structure were built on the German system; many professors and numerous doctors completed their training in Germany. Koyanagi passed away in 1954 and was buried in Sendai, after having been dissuaded from his wish to have his ashes spread in the Bay of Matsushima, off Sendai

After the inaugural article by Alfred Vogt in 1906 [1], several patients with VKH were described in Japan. The first was published by Professor Jujiro Komoto in 1911 (in German) [8]. In 1914, Yoshizo Koyanagi described 2 cases published in the Japanese journal, Nippon Ganka Gakkai Zashii [9]. However, the ground-breaking article was published by Yoshizo Koyanagi in the German journal, Klinische Monatsblätter für Augenheilkunde, in 1929. That report described 16 cases of VKH [10]. The importance of that article lay in the precise description of the natural course of VKH disease, but no treatment was available at that time (Fig. 1).

**Fig. 2:**
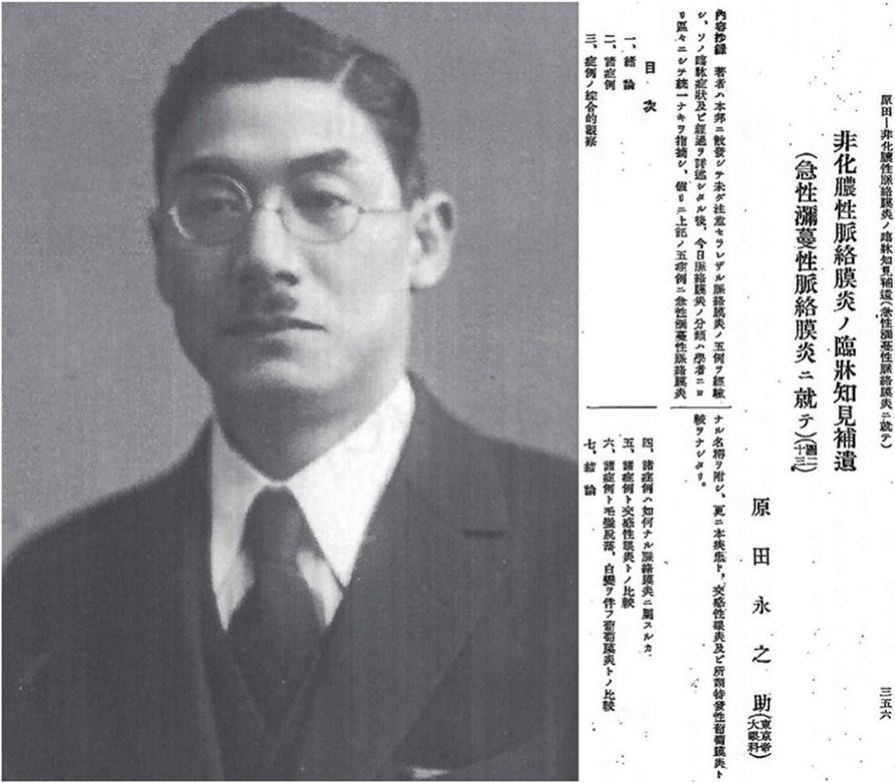
Einosuke Harada (1892–1946). Einosuke Harada was born in 1892 in Amakusa, on Kyushu Island, in Southern Japan. He married the daughter of the head of a notorious eye hospital, the Hara Eye Clinic, in Nagasaki. After training as an internist, and working for the army, Harada then reoriented his training towards ophthalmology, from 1923 to 1925 at Tokyo Imperial University. In 1923, he published the first “Harada” case, described as “acute diffuse choroiditis”. In 1926, he wrote his main article on five cases, which included the previously described case, and thus, contributed his name to the present eponym of the disease. Essentially, he described the posterior features of VKH disease. He worked in the Hara Eye Clinic in Nagasaki until 1943, when he was drafted by the army to the battlefront in the Philippines. From there, he was sent home, due to illness. His clinic was destroyed by the atomic bomb. He was planning to rebuild it but succumbed to illness in December 1946. He is buried in his hometown of Amakusa, south of Nagasaki. To the right of his portrait, the title page of his 1926 article is shown; it was published in Nippon Ganka Gakkai Zashii

In parallel, Einosuke Harada described one case in 1923 [2], published in German, and 5 cases in 1926, published in Nippon Ganka Gakkai Zashii, of a novel condition. The latter article described the posterior involvement of VKH disease, which he called acute diffuse choroiditis (choroiditis diffusa acuta; Fig. 2) [11].

The final eponym was coined by Professor Jean Babel of Geneva, who joined the names Vogt and Koyanagi in a proposal to call the disease Vogt-Koyanagi syndrome (disease). At that time, it was known that Harada’s disease and Vogt-Koyanagi syndrome were nearly identical [12]. By 1970, the majority of articles published used the term Vogt-Koyanagi-Harada disease (syndrome), and by 2003, most authors had adopted the term Vogt-Koyanagi-Harada disease [13].

## Advances in immunopathogenic mechanisms

The exact etiology of VKH disease is unknown. However, T cell-mediated autoimmune responses against melanocyte-related antigens are thought to be involved in VKH development. Several studies showed that peripheral blood mononuclear cells (PBMCs) from patients with VKH disease recognized peptides derived from the tyrosinase family of proteins (TYR, TRP1, and TRP2) which participate in melanin synthesis [14–17]. When mice were immunized with tyrosinase-related peptides, ocular inflammation was induced, which resembled VKH [18].

T cells initiate an immune response when they recognize antigen-derived peptides bound on major histocompatibility complex (MHC) molecules (also known as human leukocyte antigens [HLA]). The two primary types of MHC/HLA molecules involved in antigen presentation are known as MHC class I and II molecules. MHC class I molecules principally present peptides that are synthesized intracellularly, and they present them to T cytotoxic (Tc) cells. In contrast, MHC class II molecules present peptides derived from proteins taken up and degraded by the cell, and they present them to T helper (Th) cells [19]. Immunohistochemical studies of eyes with VKH revealed that T cells infiltrated into the choroid, and MHC class II molecules were found on choroidal melanocytes and on the endothelium of the choriocapillaris [20]. Although every human inherits a set of genes for MHC class I and II molecules, remarkable variability exists in each individual’s MHC type. Several studies have indicated that MHC class II molecules, HLA-DRB1*0405 and DRB1*0410, were robustly related to VKH susceptibility [21]. That finding suggested that peptides derived from tyrosinase-family proteins preferentially bind to the HLA-DRB1*0405 and DRB1*0410 molecules, and they are presented at the cell surface of antigen-presenting cells [16, 17]. Among the Th-cell subsets that recognize MHC class II-associated antigens, Th1 cells produce interferon-gamma [16, 17] and Th17 cells produce IL-17 [22–24]. It was shown that Th1 and Th17 cells might be involved in pathological changes associated with VKH disease, such as choroidal stroma inflammation in the acute phase of VKH. In contrast, regulatory T cells, which produce IL-10 and TGF-β, were associated with the resolution of active inflammation in VKH [25, 26].

## Clinical considerations and the distinction between initial-onset and chronic VKH

In VKH, inflammation starts exclusively in the choroidal stroma. This inflammation is triggered by an autoimmune reaction against melanocyte-associated antigens [27]. The pathogenesis of autoimmune diseases is complex and can manifest with significant differences among patients. Moreover, a comprehensive review on refractory rheumatoid arthritis suggested that treatment-resistance may arise from inflammatory or non-inflammatory mechanisms, and that these mechanisms lead to differential responses to disease-modifying anti-rheumatic drugs and outcomes. Those findings established that there were two different courses of rheumatoid arthritis [28]. In addition, other pathologies such as juvenile idiopathic arthritis and multiple sclerosis, have different clinical courses, and the therapeutic approach is different in each case [29, 30]. Consequently, making a clear distinction between different disease courses is fundamental to understanding and managing the disease [28]. Accordingly, two different VKH phenotypes were found in the literature: one was called initial-onset VKH and the other was called chronic VKH. These two subtypes showed remarkably different responses to treatment [31, 32]. Therefore, for VKH management, a clear distinction between initial-onset and chronic disease is a primary criterion for tailoring treatment.

Recent studies have provided compelling evidence on successful treatments for initial-onset VKH. Those studies represented a critical milestone in achieving disease resolution. For example, among patients with VKH that displayed an impaired response to corticosteroids, immunomodulatory therapy was beneficial, and it produced the greatest impact on visual acuity when patients started the treatment early after VKH onset [33]. Moreover, mycophenolate mofetil effectively improved choroid and optic nerve head blood flow in patients with initial-onset VKH without anterior inflammation [34]. Additionally, in patients that did not develop a sunset glow fundus, immunomodulatory therapy resolved the retinal oxygenation issues and blood vessel lumen changes that commonly occur in initial-onset VKH. However, in patients that developed a sunset glow fundus and chorioretinal atrophy, immunomodulatory therapy did not alter impaired oxygen saturation [35–37]. Those studies have shown that detecting VKH at initial onset constituted a prognostic factor for a good response to immunomodulatory therapy.

Accurate discrimination between initial-onset and chronic-recurrent VKH requires a well-defined set of criteria. When applied, these criteria might provide some insight on how to differentially treat the two disease courses. Thus, a system of diagnostic criteria was developed and tested in a cohort of Chinese patients with VKH. Those criteria yielded a substantial improvement in sensitivity and negative predictive value, compared to the Revised Diagnostic Criteria for VKH disease from 2001 [38]. Moreover, based on those criteria, early- and late-phase VKH were recognized as clinically different conditions, which required different ancillary tests, such as fluorescein angiography (FA) and enhanced depth imaging optical coherence tomography (EDI-OCT), for evaluations of inflammation-related findings [38]. A recent review (2021) on the appraisal and management of initial-onset VKH [39] introduced a new means of assessing choroiditis: indocyanine green angiography (ICGA), and EDI-OCT as a surrogate. Thus, ICGA was added to the list of proposed criteria for diagnosing VKH.

A review by Herbort et al. also recommended the combined use of corticosteroids and non-steroidal immunosuppression for the management of initial-onset VKH [39, 40]. Additionally, dual corticosteroid and immunomodulatory therapy was shown to be successful in managing VKH; indeed, most patients remained in remission, even after discontinuing treatment [39]. Lately, these ancillary tests have been considered highly important for performing a complete clinical evaluation and for determining individually-tailored treatments for reducing the risk of inflammatory recurrences.

## Description of initial-onset and chronic VKH disease

Prodromal findings of VKH can be neurological, auditory, and head skin dysesthesia manifestations. The symptoms include fever, meningismus (headache, malaise, nausea, stiffness of the neck and back), audio-vestibular manifestations (sensorineural hearing loss, tinnitus, aural fullness, and vertigo), hyperesthesia of the scalp or orbital pain [6, 41]. These symptoms anticipate ocular disease, and they arise from a combination of choroidal and meningeal inflammation, which can only be detected with ICGA [42] and a CSF evaluation [43]. This stage lasts for a number of days, but it is not always present [41].

VKH inflammation starts solely in the choroidal stroma [32]. Clinical manifestations occur when choroidal inflammation has disseminated toward the surrounding tissues, such as the optic disc, retina, ciliary body, and anterior chamber. In the initial-onset of acute VKH, bilateral panuveitis develops, mainly with serous retinal detachment, papillitis, and mild to moderate vitritis. When this condition is not treated, it progresses to involve the anterior segment, and changes in the ciliary body may cause a shallow anterior chamber [44, 45].

VKH occurs bilaterally; however, the effects may be asymmetric [46]. For instance, a unilateral retinal detachment that occurs at onset can evolve into bilateral retinal detachment, when not appropriately treated [47]. Prompt detection of the disease with adequate choroidal evaluation is fundamental. ICGA was shown to identify choroidal granulomas in 100% of patients with initial-onset acute VKH [48]; alternatively, similar efficacy is probably achievable with EDI-OCT. When classical signs are present, inflammation can be found with various modalities, including laser flare photometry, FA, EDI-OCT, and ICGA [49].

One study, based on FA and OCT, classified VKH serous retinal detachments (SRDs) into two types: a clinical SRD and an optic-disc swelling type (OD-SRD). The OD-SRD type is very mild, with no subretinal fluorescein pooling. OD-SRD type VKH was associated with older age, longer times to treatment, and a higher likelihood of developing chronic disease. The pathological mechanism of OD-SRD is related to aging, which reduces the choriocapillaris density and changes disc morphology. Therefore, FA can anticipate the course of the disease and facilitate treatment planning [50].

The therapeutic “window of opportunity” for VKH [45] is 2 to 4 weeks after onset. When treatment is not initiated appropriately, the disease will progress to chronic VKH. Chronic VKH differs from initial-onset VKH, in terms of the clinical signs, progression, treatment outcome, and the incidence of complications [40]. However, despite early administration of high-dose corticosteroids in the initial acute stage, 22.5% to 79% of patients can develop chronic VKH.

Chronic VKH is often characterized by a sunset glow fundus, and it occurs after 2–6 months. Among patients with chronic VKH, 38% progress to subretinal fibrosis [50–53]. Mild inflammation often persists, even when systemic steroid therapy is properly administered in the early stage of onset, and after the inflammation seems to be resolved. Exacerbations of VKH often occur in the form of chronic recurrent granulomatous anterior uveitis. Importantly, a significant correlation was found between anterior chamber activity and choroidal thickening, which may represent subclinical inflammation at the level of the posterior segment [6, 41, 54]. In those cases, ICGA may show evidence of choroidal inflammation (e.g., hypofluorescent dark dots), an indication that treatment adjustments should be considered.

In chronic VKH disease, depigmentation of the choroid makes the fundus appear red, known as a sunset glow sign. This sign appears in 60–70% of patients, and it appears more commonly in Asian patients [41]. Additionally, the atrophy of cells around the fundus causes a white patchy appearance, which is accompanied by local depigmentation of retinal pigment epithelial (RPE) cells. This feature is often confused with Dalen-Fuchs nodules, but the Dalen-Fuchs nodule is a granuloma pathologically formed by RPE and inflammatory cells. Consequently, the patchy atrophic lesions observed in the fundus are called scars of Dalen-Fuchs-like nodules [55]. Chronic VKH is also associated with complications that threaten vision, like cataract, glaucoma, choroidal neovascular membranes, subretinal fibrosis, and chorioretinal atrophy [41, 54, 56]. Accordingly, various studies have reported that chronic VKH was associated with poor visual acuity and reduced retinal sensitivity [44, 57].

## Imaging methods for diagnosing and monitoring VKH disease

The appraisal of inflammation in posterior uveitis with imaging has made significant advances since the 1990s. However, modalities immediately relevant for the diagnosis and follow-up of stromal choroiditis must be distinguished from modalities that are interesting for investigational research, but ill-adapted and/or insufficiently standardized for diagnostic and/or follow-up purposes. A selection of new imaging modalities may be technically very elaborate, but inappropriate for investigating structures that they were not designed to image.

**Fig. 3:**
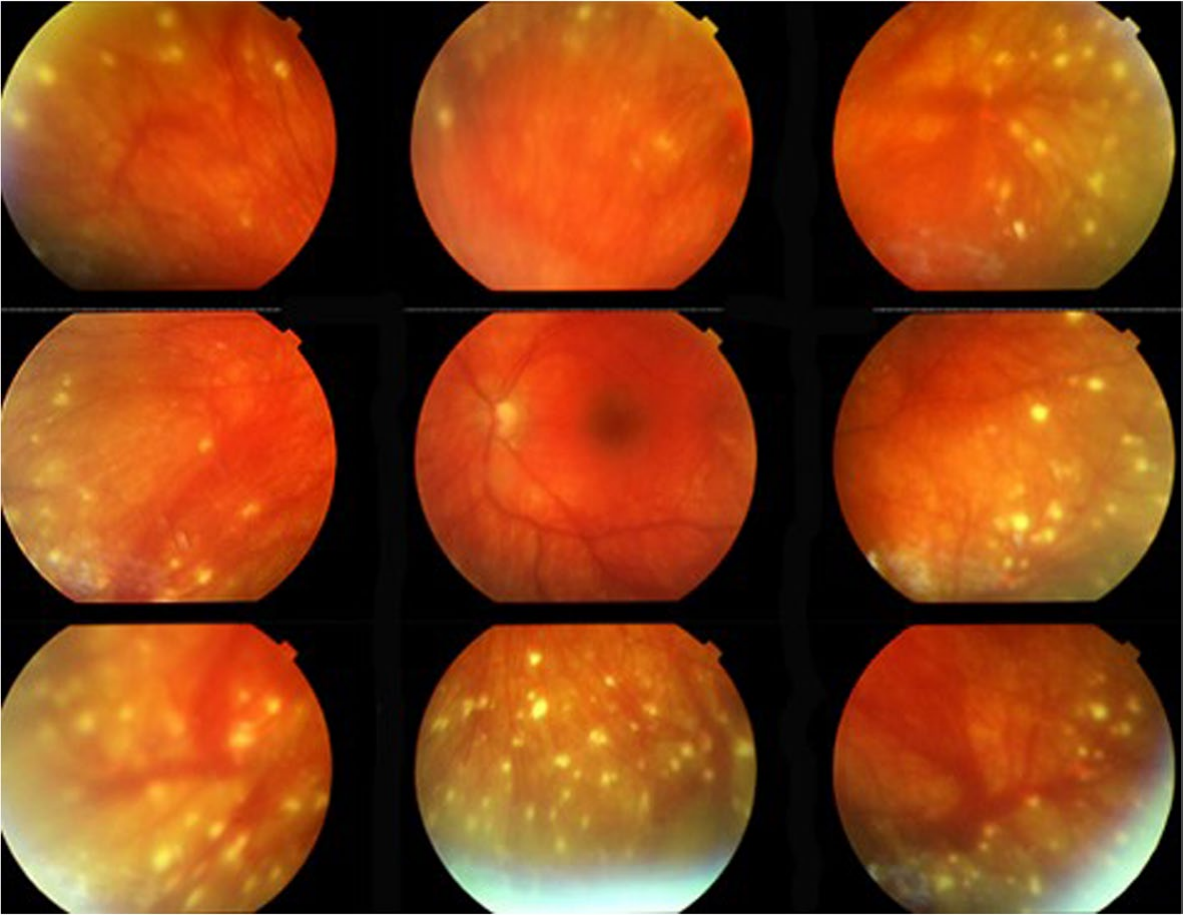
Fundus findings in chronic recurrent VKH. The eye of a north-African patient is shown, with a sunset glow fundus and numerous scars of Dalen-Fuchs nodules

Fundus photography is part of the routine imaging work-up for VKH disease. In acute disease, fundus photography shows exudative retinal detachments. During follow-up in chronic disease, fundus photography shows Dalen-Fuchs like nodules, and it is crucial in portraying depigmentation of the fundus (i.e., a sunset glow fundus; Fig. 3) [58, 59].

### Fluorescein angiography

**Fig. 4:**
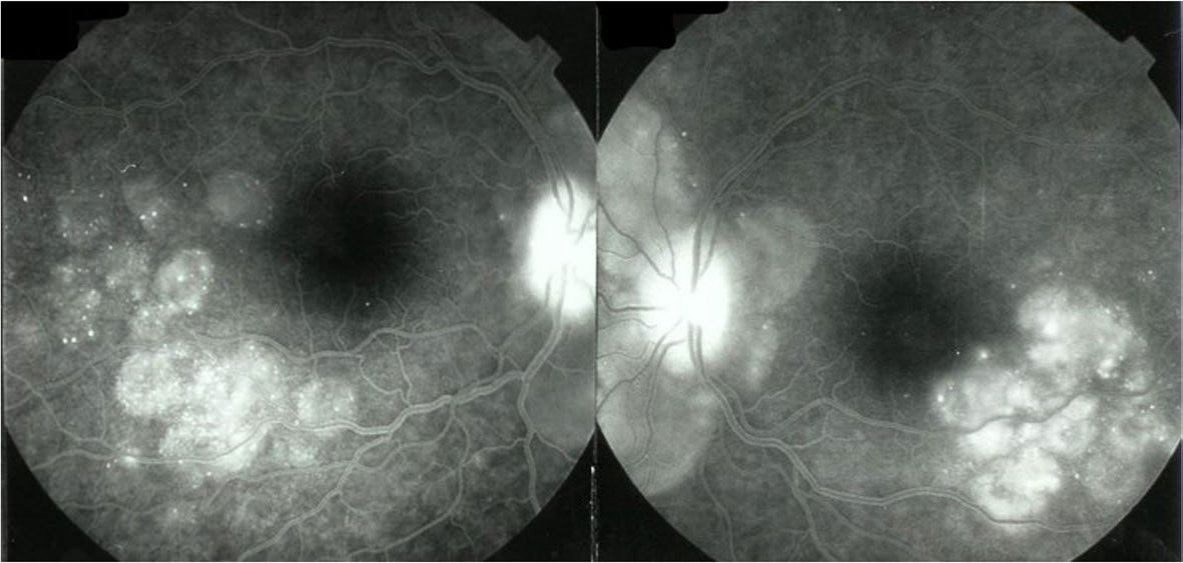
Fluorescein angiogram findings in acute phase VKH. Hyperfluorescent pinpoints and pooling mark the exudative retinal detachments. Note the bright hyperfluorescent disc

Fluorescein angiography (FA) cannot show choroidal inflammation. However, in the acute exudative phase, FA can show spill-over inflammation which extends from the choroid to the retina and the optic disc. Recent diagnostic criteria for initial-onset VKH include two FA findings: exudative retinal detachment and disc hyperfluorescence [39, 60]. Characteristic signs include, initially, focal areas of delay in choroidal perfusion and choroidal folds, which appear as long, hypofluorescent lines that radiate from the optic nerve. Subsequently, multiple hyperfluorescent pinpoints and progressive subretinal pooling delineate exudative retinal detachments. These conditions lead to disc hyperfluorescence (Fig. 4) [61, 62] and vascular hyperfluorescence [63].

Peripapillary hyperfluorescent pinpoints can be observed in the hyperacute phase. Failure to observe this sign may imply that the FA was performed at a later stage, and thus, a more aggressive, prolonged regimen of immunosuppressive therapy would be required [64]. Additionally, hyperfluorescent lesions detected with FA were correlated with RPE detachments detected with spectral domain optical coherence tomography (SD-OCT) [65].

A recent study with ultra-wide field FA (UWF-FA) identified several uncommon features of VKH in the central and peripheral retina. Those results indicated that focal leakage occurred in 92.3%, pooling with a dark rim occurred in 84.6%, and vasculitis occurred in 46.2% of patients with VKH. Overall, UWF-FA detected 76.9% of the abnormal findings that could be identified with ICGA. Peripheral vascular leakage arises from choroidal inflammation. Thus, UWF-FA provides more data than conventional FA, and it can be used to evaluate responses to treatment and prognosis [66].

**Fig. 5:**
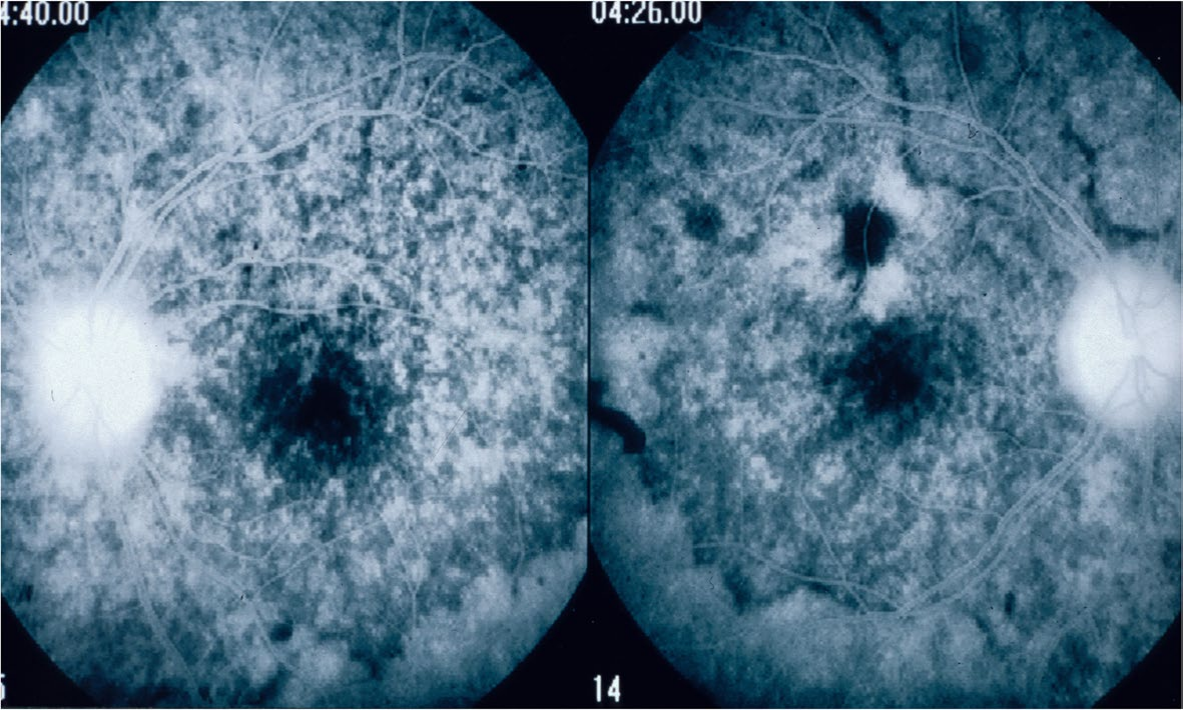
Fluorescein angiogram findings in the chronic stage of VKH disease. Hyperfluorescent areas (window-defect) and hypofluorescent areas (pigment clumps) are visible in the posterior pole. Note the high-water marks, which indicate the extent of exudative retinal detachment. Note also the bright hyperfluorescence of both discs

In the chronic phase of VKH, FA shows window defects in areas where RPE cells are lost, or a fluorescence-blocking effect in areas of pigment clumping from destroyed RPE cells. FA can also clearly show the limits of exudative detachments during the acute phase after reattachment (high water marks), which appear as hyperpigmented lines (Fig. 5) and disc hyperfluorescence [64]. Another sign of scarring is the appearance of dot-like, equatorial hyperfluorescence [63].

FA can facilitate the diagnosis of several complications, including arteriovenous and retinochoroidal anastomoses, optic disc neovascularization, and choroidal neovascularization [63].

### Optical coherence tomography

SD-OCT is an advantageous imaging modality, because it is non-invasive; therefore, it can be repeated without risk. In its normal mode (retinal OCT), it allows the visualization of sections through the different retinal layers up to the RPE. In 2008, EDI-OCT was first described. EDI-OCT provides an image of the choroid and, in particular, the ability to measure choroidal thickness [67]. The current instruments used in clinical practice are limited, because they can only image the posterior pole. This is not a problem, when analyzing acute exudative retinal detachments in VKH, which occur mostly in the posterior pole. However, it represents a handicap for monitoring occult choroidal inflammation; thus, ICGA is the more appropriate modality for obtaining information on the entire fundus [68].

### Retinal SD-OCT

**Fig. 6:**
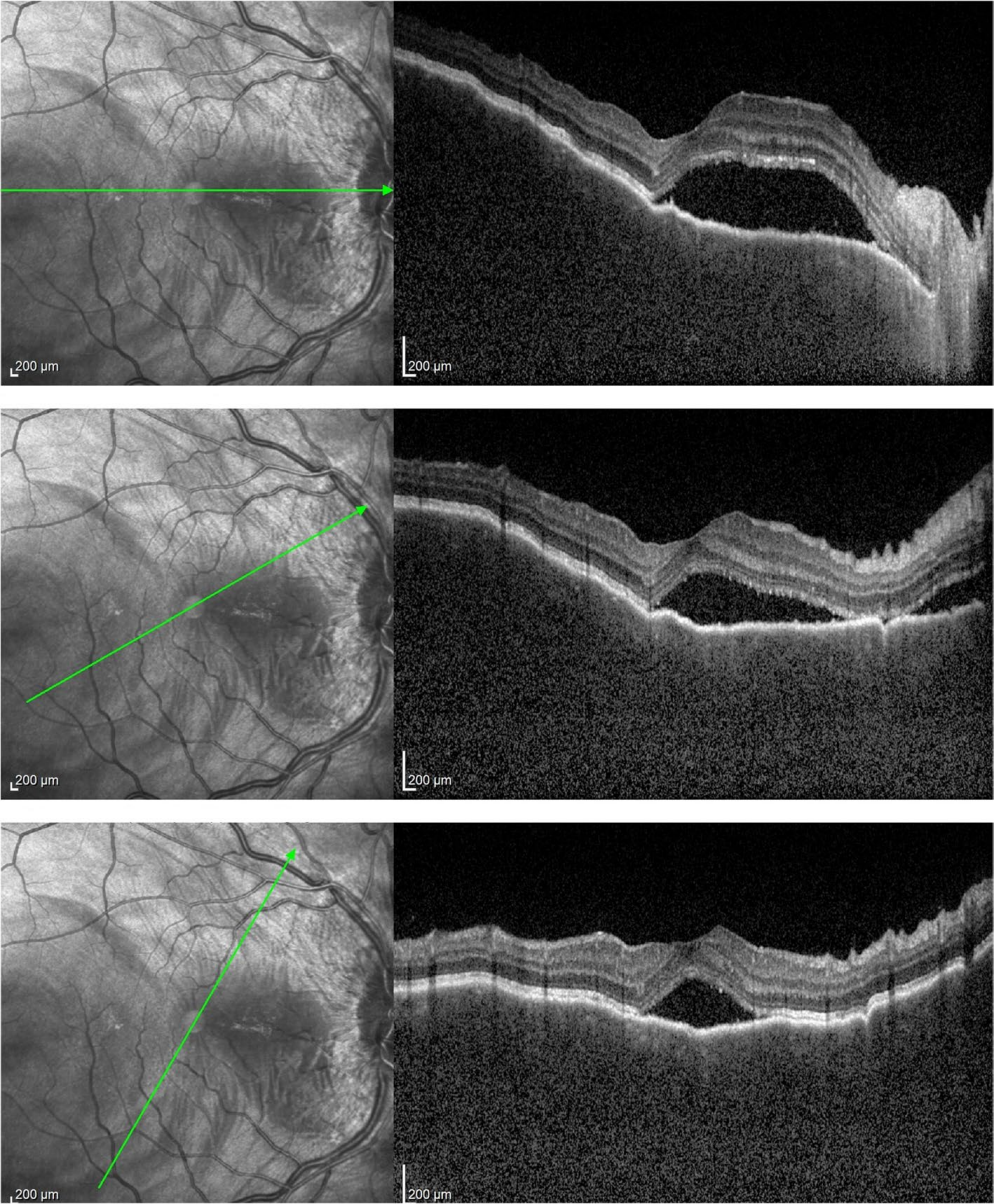
SD-OCT findings in the acute phase of VKH. Choroidal folds and exudative retinal detachments that split the photoreceptor layer

In acute initial-onset VKH disease, the advent of SD-OCT dramatically increased the precision of analyzing exudative retinal detachments. In 2004, Maruyama and Kishi distinguished two types of SRDs: a true, complete retinal detachment on one side, and detachments that separated the outer retinal layers from the intraretinal fluid [69]. Later studies conducted in the same institution showed that the multi-lobular dye pooling detected with FA was due to subretinal septa formed from inflammatory products, such as fibrin [70]. Moreover, SD-OCT provided more precise morphological information about splitting in the photoreceptor layer in exudative retinal detachments (Fig. 6) [71]. One study clearly showed intraretinal splitting at the junction of the inner and outer segments [72]. Choroidal/RPE folds were described as a frequent sign in patients with acute initial-onset VKH disease [73, 74]. This manifestation was related to more severe disease and longstanding inflammation in choroidal tissue [74, 75].

In chronic VKH, SD-OCT could show signs of subclinical inflammation, such as macular edema and early signs of macular complications [52]. Regardless of the specific OCT findings, the most practical aspect of SD-OCT is its ability to provide close monitoring of lesion remission after (aggressive) treatment.

### Choroidal EDI-OCT

**Fig. 7:**
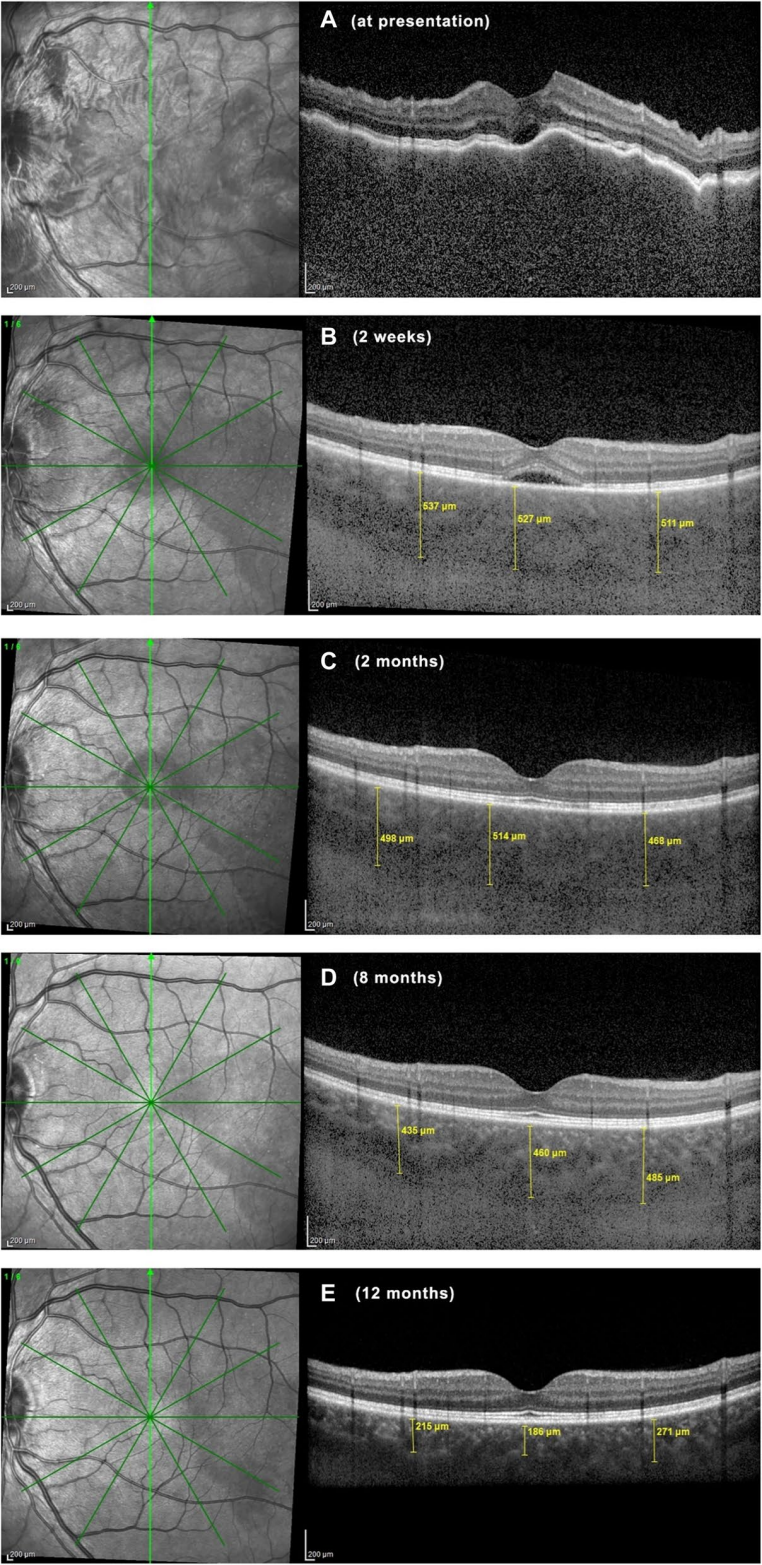
EDI-OCT findings in course of VKH, from presentation to a 12-month **follow-up. A** At presentation, the choroid is too thick to be measured (note choroidal folds); **B** after 2 weeks, the limit of the choroidal space can again be recognized, but choroidal thickness is increased (mean subfoveal thickness = 525 ± 13.1 μm); **C**-**E** the choroidal thickness progressively decreased; **C** after 2 months, the choroidal thickness was 493.3 ± 23.5 μm); **D** after 8 months, the choroidal thickness was 460.3 ± 24.5 μm; and **E** at 12 months, the choroidal compartment is thinner than normal (224 ± 43.2 μm)

EDI-OCT is a non-invasive imaging modality that makes it possible to analyze choroidal involvement in VKH. Depending on the stage of the disease, choroidal thickness is either increased (in early-stage disease) or reduced (in late-stage and/or ill-treated disease). In the early disease stage, EDI-OCT could clearly show increases in choroidal thickness (Fig. 7), and the thickness was shown to decrease progressively with treatment. However, during corticosteroid tapering, a rebound in the sub-foveal choroidal thickness was observed in some cases of recurrence [76, 77]. In the acute stage of VKH, choroidal thickness was found to decrease at the expense of the outer choroid. Consequently, it was suggested that the primary target of the disease might be located in that layer [78]. In the absence of appropriate treatment, long periods of uncontrolled disease may cause a reduction in choroidal thickness over time, due to choroidal atrophy. Several reports have shown choroidal thinning in convalescent or chronically evolving disease [79, 80]. Moreover, in the late stages of VKH, choriocapillaris layer disruptions were described [81].

EDI-OCT is a valuable diagnostic and monitoring modality for initial-onset VKH disease. It provides information on choroidal thickness, and hence, on inflammatory choroidal infiltration [76, 77]. In the very early stage of VKH, the choroid thickens in proportions that cannot be measured. With treatment, choroidal thickness progressively decreases, and this thickness is a useful parameter for monitoring disease evolution. In the subacute phase of VKH, EDI-OCT provides less precise information than ICGA, because the range of EDI-OCT is limited to the posterior pole [49, 68, 82, 83]. However, in post-acute disease, choroidal thickness can result from disease remission, in some areas of the choroid, and from reactivation, in other areas. Thus, these measurements are difficult to interpret. However, several reports have shown that EDI-OCT could indicate subclinical reactivations before they were clinically apparent [84, 85] and it could indicate atrophic sunset glow fundus evolution [86]. In addition, some authors demonstrated that the level of anterior segment inflammation was moderately correlated to the sub-foveal choroidal thickness [87]. Nevertheless, as the choroid becomes thinner, changes in the sub-foveal choroidal thickness become more variable during recurrences [86, 87].

### Assessing choroidal involvement and early VKH diagnosis

ICGA became available in the mid-1990s [88]. The advent of ICGA provided substantial progress in appraisals of posterior uveitis because it allowed precise evaluations of inflammation in the choroidal compartment. These evaluations were previously not possible or only roughly possible with echography [89]. For the first time, with ICGA, the clinicopathology of primary or secondary choroiditis entities could be clarified, due to the ability to distinguish stromal choroiditis from choriocapillaritis [90].

**Fig. 8:**
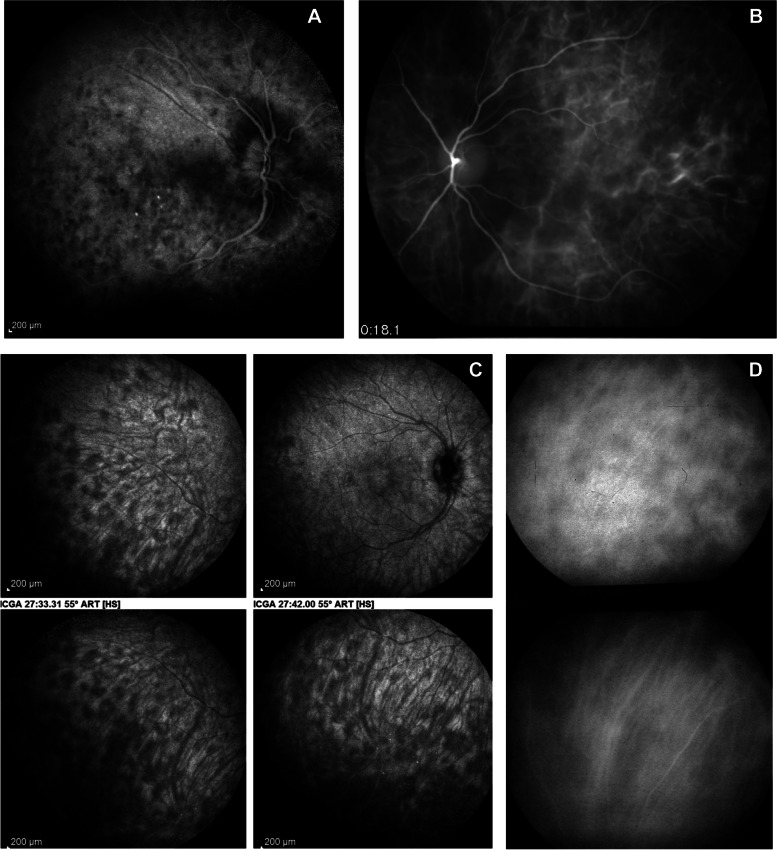
Four main signs detected with ICGA in acute initial-onset VKH disease. **A** Hyperfluorescent disc (usually hypofluorescent on ICGA), which indicates severe inflammation. **B** Early hyperfluorescent vessels. **C** Numerous, regularly distributed hypofluorescent dark dots (HDDs), evenly sized, over the whole fundus, is the most demonstrative and quantifiable ICGA sign. **D** (*top*) Fuzzy, indistinct choroidal vessels represent the fourth ICGA sign; (*Bottom*) After 3 days of intravenous methylprednisolone treatment, the course and structure of choroidal vessels are again distinctly visible

The principles of ICGA that applied to posterior uveitis were defined in 1999 [91]. Four main signs of stromal choroiditis (including VKH disease) were identified (Table 1**;** Fig. 8) [42]. Later, these findings were verified in different populations [48, 92, 93].

**Table 1:** ICG angiographic signs in stromal choroiditis

1.	Hypofluorescent dark dots (HDDs)
2.	Indistinct choroidal vessel (Fuzziness of choroidal vessels)
3.	Diffuse late choroidal hyperfluorescence (partially hiding HDDs)
4.	ICGA disc hyperfluorescence (in severe choroiditis)

ICGA is crucial for evaluating stromal choroiditis (like VKH) for several reasons, including: (1) it shows the primary lesion; i.e., inflammatory choroidal infiltration ([94], 2) it gives a global, pan-fundal overview of the stromal lesions, and therefore, it is superior to EDI-OCT (which is mostly limited to the posterior pole) [68, 82]; and (3) it allows identification of subclinical lesions, which provides (a) an early diagnosis and (b) a means to monitor the subclinical evolution of the disease [95, 96]. ICGA monitoring of subclinical choroiditis can ensure zero tolerance of inflammation after treating initial-onset disease [97]; and thus, it enables a cure for the disease in a substantial proportion of patients [4, 37]. Consequently, ICGA is clearly the gold standard for appraisals and follow-ups in VKH disease, however EDI-OCT should be especially considered in those geographic areas where ICGA is not fully available.

### Additional imaging methods for diagnosing and monitoring VKH disease

Recently, new imaging methods have been applied to VKH disease that are of some interest in research, but they are not directly relevant for practical diagnostic and follow-up purposes. These methods are either not standardized, or they are not appropriate, mainly because they were not designed to provide information on choroidal stroma. The two modalities that have been applied to many clinical entities, including VKH, are blue light fundus autofluorescence (BL-FAF) [98] and OCT-angiography (OCT-A) [99].

BL-FAF is very useful for assessing diseases that involve the RPE, the photoreceptors, or the choriocapillaris. However, VKH does not primarily involve the RPE or choriocapillaris. These structures are involved secondarily, due to underlying choroidal swelling and overlying exudative detachments. Thus, BL-FAF findings do not directly reflect the morphological changes caused by VKH; instead, those findings depend on the severity of choroiditis or exudative detachment [100]. Consequently, BL-FAF cannot be used for routine assessments, unlike OCT and ICGA, which provide direct assessments of primary lesions. BL-FAF findings are difficult to interpret because the lesions depend on the stage and evolution of the disease, and they are not essential for routine practical purposes. However, BL-FAF can provide information on the disease stage and disease severity, particularly in chronic disease.

We identified less than five studies on VKH in the PubMed database that used “OCT-A” in the title [99]. OCT-A can detect secondary choriocapillaris/RPE loss and inflammatory choroidal neovascularization. Thus, both BL-FAF and OCT-A are useful for documenting potential secondary evolutions of the disease, but not for routine primary systematic evaluations of VKH disease intensity or follow-up. Accordingly, although it is an overstatement to speak of the role of OCT-A in the diagnosis and management of VKH, its value lies in documenting some of the consequences of stromal choroiditis on neighboring structures.

## Practical diagnostic criteria for early VKH diagnosis

With the availability of effective inflammation-suppressive treatment [101, 102], it became necessary to develop systematized, effective, diagnostic criteria to ensure that therapy could be introduced as soon as possible. In 1978, Seiji Sugiura’s VKH diagnostic criteria were published in the English language [103]. Thereafter, in 1980, a new definition of VKH diagnostic criteria was attempted in a country (United States) where the incidence of VKH was rare [104]. In 1999, a workshop was held under the auspices of the University of California and the University of Southern California with the aim of revising the diagnostic criteria for VKH [5]. However, these two sets of criteria were insufficient, due to the lack of sensitive investigative procedures for choroiditis and the inadequate separation between initial-onset and chronic forms of the disease.

In the 1990s, the development of ICGA made it possible to image the choroidal compartment, which enabled precise, reliable assessments of choroidal inflammation [42]. Because VKH is primarily a choroidal inflammatory disease, ICGA substantially improved the appraisal of VKH disease. ICGA provided unparalleled sensitivity for diagnosing, monitoring, and following the evolution of VKH disease [90]. Recently, more precise criteria were defined and we moved one step closer to establishing adequate, simple diagnostic criteria for VKH. These criteria took advantage of novel, sensitive imaging methods for investigating the choroid [39]. Importantly, separate criteria were defined for initial-onset (Table 2) and chronic VKH disease (Table 3).

**Table 2:** Diagnostic criteria for initial-onset acute Vogt-Koyanagi-Harada disease^a^

1.	No ocular trauma or surgery preceding onset of disease^b^
2.	Bilateral involvement (verified with ICGA and/or EDI-OCT)^b^
3.	Exclusion of other infectious, inflammatory or masquerading entities, in particular other stromal choroiditis entities (i.e., tuberculosis, sarcoidosis or syphilis)^b^
4.	Diffuse choroiditis evidenced by ICGA and/or EDI-OCT^b^
5.	Signs and symptoms of less than 4 weeks’ duration^b^
6.	Absence of clinical findings compatible with chronic disease (i.e. sunset glow fundus or integumentary signs (vitiligo, alopecia & poliosis)^b^
7.	Exudative retinal detachments (evidenced by pooling and pinpoint leaking points on FA and ICGA) (very helpful criterion when present)
8.	Disc hyperfluorescence (helpful criterion).
9.	Neurological / auditory findings (meningismus, tinnitus, acute hearing loss) (helpful criterion).

^a^Reprinted from: Herbort CP Jr., et al. [39]

^b^Essential criteria

**Table 3:** Diagnostic criteria for chronic recurrent Vogt-Koyanagi-Harada disease

1.	No ocular trauma or surgery preceding onset of disease^a^
2.	Bilateral involvement (verified with ICGA and/or EDI-OCT)^a^
3.	Exclusion of other infectious, inflammatory or masquerading entities, in particular other stromal choroiditis entities (i.e. tuberculosis, sarcoidosis or syphilis)^a^
4.	Signs and symptoms of more than 4 weeks´ duration before treatment initiation^a^
5.	Ocular depigmentation findings (i.e., sunset glow fundus, Sugiura sign, Dallen-Fuchs-like nodules)
6.	Integumentary findings (i.e., vitiligo, poliosis)
7.	Choroidal atrophy (evidenced by thinning and/or distortion of vascular architecture on EDI-OCT and/or ICG)
8.	Treatment refractoriness, defined as absence of inflammatory improvement or persistence of inflammation (namely not achieving a two-step decrease in the level of inflammation or a decrease to grade 0 and/or persistence of retinal detachment), despite a minimum of 4 to 6 weeks of systemic therapy [105]
9.	Presence of ocular complications, such as cataract, glaucoma, hypotony, choroidal neovascularization.
10.	Presence of granulomatous anterior segment inflammation (namely Koeppe/Bussacca iris nodules plus mutton-fat keratic precipitates) [6, 44].

^a^Essential criteria

## VKH management

In recent years, VKH treatment paradigms have evolved substantially. Koyanagi’s groundbreaking publication precisely described the natural evolution of the disease in the absence of treatment [10]. When corticosteroid treatment became available in the 1950s [101, 102, 106, 107], the perception of VKH evolution was modified from a non-treatable to a chronically evolving disease. After that, it became clear that two forms of VKH should be distinguished, the initial-onset disease and the insufficiently treated chronic disease. Indeed, in some cases, corticosteroid therapy provided a cure, but in a large proportion of cases, corticosteroid therapy could not completely cure the disease or avert chronic evolution [53]. An increasing number of clinicians suggested that additional, non-steroidal immunosuppressive agents were needed [108, 109].

This review was not designed to describe in detail the new approaches for VKH treatment, which have been described recently [31, 39]. Instead, here, we summarize the following general principles:

1. Corticosteroid monotherapy was shown to be insufficient in arresting the disease, even when given early and in high doses [53].
2. A combination of steroidal and non-steroidal immunosuppression, as first-line therapy, could prevent chronic evolution and cure the disease [37]. In a study that included 974 patients, corticosteroid monotherapy was compared to combined steroidal and non-steroidal immunosuppression. They found that chronic evolution was reduced from 44% to 2.3% in the combined treatment group [4].
3. Early treatment is another pre-requisite for successful management of VKH [32, 110]. It is crucial to treat within the therapeutic window of opportunity. The window of opportunity for initial-onset VKH is between 2 and 4 weeks, but it is probably different for each patient. Studies that aim to determine a more precise time interval are presently ongoing.
4. Therapy should aim for zero tolerance of choroidal inflammation, monitored with ICGA or EDI-OCT [95, 97].
5. Because efficient therapy can vary from patient to patient, therapies are administered in trial-and-error mode, facilitated by closely monitoring the evolution of choroidal lesions with ICGA [82].

## Conclusion

At 115 years after the first description of VKH, we currently understand its course and behavior. We can diagnose it early, treat it efficiently, and monitor it precisely.

## Data Availability

Not Applicable.

## Abbreviations

BL-FAF: Blue light fundus autofluorescence
EDI-OCT: Enhanced depth imaging OCT
FA: Fluorescein angiography
HLA: Human leukocyte antigens
ICGA: Indocyanine green angiography
IL: Interleukin
MHC: Major histocompatibility complex
OCT: Optical coherence tomography
OCT-A: OCT-angiography
OD-SRD: Optic-disc swelling SRD
PBMCs: Peripheral blood mononuclear cells
RPE: Retinal pigment epithelium
SD-OCT: Spectral domain OCT
SRDs: Serous retinal detachments
Tc: T cytotoxic cells
TGF-β: Transforming growth factor β
Th: T helper cells
TRP: Tyrosinase-related proteins
TYR: Tyrosinase
UWF-FA: Ultra-wide field FA
VKH: Vogt-Koyanagi-Harada
